# Cardiovascular disease risk in early‐onset vs late‐onset type 2 diabetes in China: A population‐based cross‐sectional study

**DOI:** 10.1111/1753-0407.13493

**Published:** 2023-11-06

**Authors:** Fei Chen, Liping Yu, Shuo Xie, Zhaoqing Li, Ruifen Deng, Xian Jin, Yifan He, Zhaojun Yang, Yao Wang, Wenying Yang, Bo Zhang, China National Diabetes and Metabolic Disorders Study Group

**Affiliations:** ^1^ Department of Endocrinology China‐Japan Friendship Hospital Beijing China; ^2^ China‐Japan Friendship Hospital (Institute of Clinical Medical Sciences) Chinese Academy of Medical Sciences & Peking Union Medical College Beijing China

**Keywords:** China National Diabetes and Metabolic Disorders Study, cross‐sectional study, early‐onset type 2 diabetes, nonfatal cardiovascular disease

## Abstract

**Background:**

This study investigated the effects of early‐onset type 2 diabetes (EOD) vs late‐onset type 2 diabetes (LOD) on nonfatal cardiovascular diseases (CVD) in China.

**Methods:**

We conducted a cross‐sectional survey of 46 239 participants from 14 provinces in China from 2007 to 2008, selecting 4949 participants with type 2 diabetes for analysis. Participants were categorized as EOD (<40 years) or LOD (≥40 years) based on age at diabetes diagnosis. Sociodemographic and nonfatal CVD information was collected through an interviewer‐assisted questionnaire and clinical examination. Logistic regression analysis was used to investigate the nonfatal CVD risk.

**Results:**

Out of 4949 participants with type 2 diabetes, 390 (7.88%) had nonfatal CVD. Participants with EOD had a higher age‐standardized prevalence of nonfatal CVD than those with LOD (11.4% vs 4.4%). Compared to LOD patients, EOD patients tended to be males and had a higher family history of diabetes, unhealthy lifestyle behaviors, and lower blood pressure levels. After adjustment for age and sex, EOD patients had a higher risk of nonfatal CVD than LOD patients (odds ratio [OR] 2.3, 95% CI 1.5–3.5). After further adjustment for diabetes duration, use of drugs, and other risk factors, the OR of nonfatal CVD was reduced but significant (OR 1.8, 95% CI 1.1–2.9). Sensitivity analysis revealed that EOD patients with metabolic syndrome had an increased nonfatal CVD risk compared to LOD patients (OR 2.0, 95% CI 1.2–3.5).

**Conclusions:**

EOD patients are at increased risk of nonfatal CVD. Individualized intervention and management measures for EOD patients are necessary.

## Graphical abstract

**Highlights**

- The study emphasizes the importance of implementing individualized prevention, intervention, and management strategies tailored to early‐onset type 2 diabetes patients to address their heightened risk of nonfatal cardiovascular diseases.
- Early‐onset type 2 diabetes patients in China have a significantly higher age‐standardized prevalence of nonfatal cardiovascular disease compared to late‐onset type 2 diabetes patients.
- Early‐onset type 2 diabetes patients tend to exhibit risk factors such as male gender, higher family history of diabetes, and unhealthy lifestyle behaviors (smoking, alcohol consumption, irregular physical activity), which contribute to their increased risk of nonfatal cardiovascular diseases.
- Early‐onset type 2 diabetes with metabolic syndrome have an increased nonfatal cardiovascular disease risk compared to late‐onset type 2 diabetes patients.

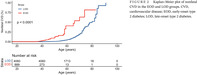

## 1 BACKGROUND

In recent decades, rapid economic development and lifestyle changes in China have led to a significant increase in the prevalence of diabetes.^1^, ^2^, ^3^, ^4^, ^5^ The number of individuals with diabetes in China is 116.6 million, which is estimated to rise to 147.2 million by 2045.^6^ Type 2 diabetes, which accounts for 95% of all diabetes cases,^7^ has been conventionally considered a disease of middle and older age. However, in recent years, a higher proportion of early onset‐type 2 diabetes (EOD, defined as type 2 diabetes diagnosed before the age of 40 years^8^) has been observed in China. The prevalence of EOD was 5.7% in 2013,^4^ and it has been increasing over the last decade.^3^, ^9^ This trend indicates that EOD poses a significant health burden in China.

Previous studies have reported that EOD is associated with poor metabolic control and accelerated development of complications.^10^, ^11^, ^12^ One study showed that EOD significantly increases the risk of cardiovascular and renal complications compared to patients with late‐onset type 2 diabetes (LOD) of similar ages.^13^ Another study conducted in China found that EOD increases the risk of nonfatal cardiovascular disease compared to LOD in hospitals.^14^ However, these studies are based on samples from either hospitals or certain regions, which may introduce selection bias. It is important to be cautious when applying the results of studies to the general population.^15^ Therefore, it is necessary to compare the nonfatal cardiovascular disease (CVD) risk with the EOD and the LDO in representative populations.

This study aimed to investigate the risk of non‐fatal CVD in patients with EOD and LOD using population‐based data. Additionally, we examined the nonfatal CVD risk in EOD vs LOD with metabolic syndrome to investigate the consistency of result.

## 2 METHODS

### 2.1 Study population

**FIGURE 1:**
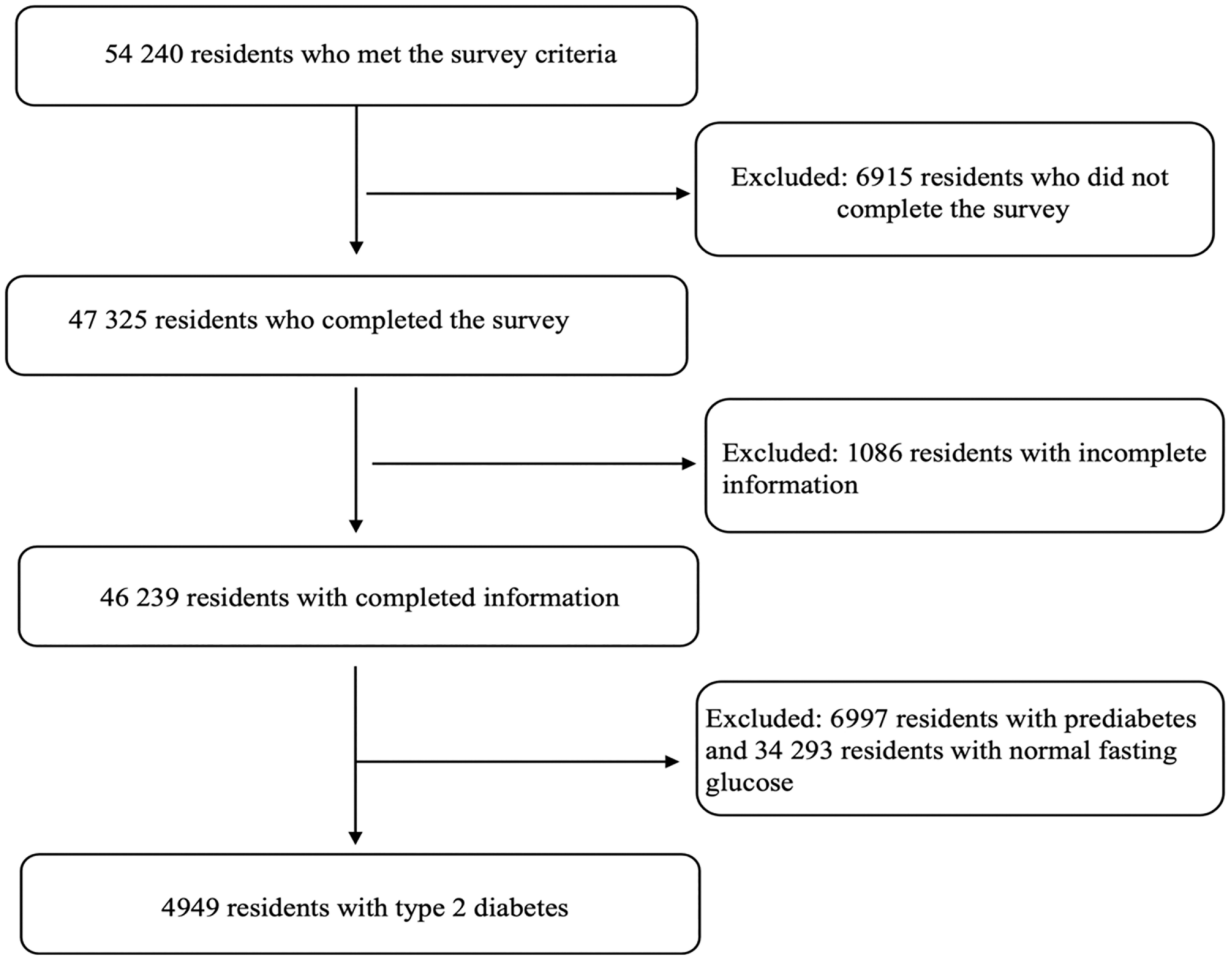
Flow chart of the study population.

This study utilized data from the China National Diabetes and Metabolic Disorders Study, a cross‐sectional study conducted between June 2007 and May 2008. A multistage stratified sampling design was employed, selecting representative cities and countries based on geographical distribution, economic development, and urbanization 2. Participants aged 20 years and above, residing in their respective areas for at least 5 years, were randomly selected. Participants who met any of the following criteria were excluded: (a) those who did not complete the survey, (b) those with incomplete information, and (c) those who had prediabetes or normal fasting glucose. Consequently, we included 4949 participants with type 2 diabetes in our analysis (Figure 1).

The present study was reviewed and approved by the ethics committee of the China–Japan Friendship Hospital, and written informed consent was obtained from each participant before data collection.

## 3 PHYSICAL EXAMINATION AND QUESTIONNAIRE

Physical examinations, including measurements of height, weight, waist circumference, hip circumference, and blood pressure were conducted following standard procedures. Hypertension was defined as an average blood pressure >140/90 mm Hg, a previous diagnosis of hypertension, or the use of antihypertensive drugs. A standardized questionnaire was completed by trained doctors and nurses, which included questions regarding family income, education level, lifestyle, family history of diseases (limited to first‐degree relatives), nonfatal CVD, and medication usage. The medications in question included hypoglycemic drugs, antihypertensive medications, and lipid‐lowering drugs. Hypoglycemic drug usage was defined as the use of medications prescribed by a healthcare provider for managing glucose levels at least once. The definitions for antihypertensive drug usage and lipid‐lowering drug usage were similar to that of hypoglycemic drug usage.

## 4 BIOCHEMICAL MEASURES AND OUTCOMES

The participants were instructed not to exercise excessively or alter their diet for at least 3 days prior to glucose testing. Overnight fasting blood samples were collected in vacuum tubes containing sodium fluoride to measure fasting plasma glucose and lipid levels. The participants who did not have a history of diabetes underwent an oral glucose tolerance test using 75 g of glucose. According to the diagnostic criterial for diabetes established by the World Health Organization in 1999, newly diagnosed diabetes was defined by fasting glucose levels ≥7.0 mmol/L, 2‐h glucose levels ≥11.1 mmol/L, or both. For safety considerations, participants who had already been diagnosed with diabetes by a physician were administered a standard meal test containing 80 g of carbohydrates. Fasting plasma glucose and lipid levels were tested in local laboratories approved by the national or provincial quality control systems.

Metabolic syndrome was defined using the National Cholesterol Education Program Adult Treatment Panel III criteria as having more than three of the following five metabolic components.^16^ We adopted the criteria of obesity, with a waist circumference ≥ 90 cm for males and ≥ 80 for females, tailored for Asian population. Central obesity was defined according to the World Health Organization criteria.^17^

Nonfatal CVDs were determined based on self‐reported provided by the participants. The specific criteria used for determining different types of CVDs were as follows: coronary heart disease (CHD) was defined as a history of hospitalization for myocardial infarction (heart attack) or a surgical history involving coronary balloon angioplasty, coronary stent implantation, or coronary artery bypass. Stroke was defined as a history of language or physical dysfunction that lasted for more than 24 h and ischemic or hemorrhagic stroke diagnosed using computed tomography or magnetic resonance imaging. Overall, nonfatal CVD referred to a history of either CHD or stroke, as defined by the aforementioned criteria.

## 5 STATISTICAL ANALYSIS

The participants were divided into two groups: EOD, including those with a diabetes diagnosed age of <40 years, and LOD, including those with a diabetes diagnosed age of ≥40 years. The baseline characteristics of diabetes were described as the mean (SD) for quantitative parameters and as a percentage for categorical parameters. Student's *t* test or 𝜒^2^ test was used to compare the difference between the two groups, as appropriate. Age‐standardized prevalence was calculated by the direct method with the use of data on the population in China in 2006.^18^ Logistic regression was to investigate the odds ratio for nonfatal CVD for EOD compared to LOD using three models. Model 1 was adjusted for age and sex; model 2 was further adjusted for smoking status, alcohol consumption, college education, regular leisure‐time physical activity, body mass index (BMI), systolic blood pressure (SBP), low‐density lipid cholesterol (LDLC), family history of hyperlipidemia, hypoglycemic drugs, antihypertensive drugs, and lipid‐lowering drugs; and model 3 was further adjusted for diabetes duration. To investigate the consistency of results, we also assessed the odds ratio of nonfatal CVD in the metabolic syndrome group.

Statistical analyses were performed using R version 4.1.0. All statistical tests were two sided, and the significance level was set at *p* < .05.

## 6 RESULTS

As shown in Table 1, 889 participants were classified as having EOD, and 4060 were classified as having LOD. Compared with the LOD group, the EOD group was more likely to be male and to exhibit unfavorable lifestyles, such as smoking, alcohol consumption, and irregular leisure‐time physical activity. Moreover, a greater family history of diabetes and hyperlipidemia was observed in the EOD group. Interestingly, fasting glucose levels were higher in the EOD group than in the LOD group; however, postprandial 2‐h glucose levels were lower. Moreover, we compared the clinical characteristics of EOD both with and without CVD, as well as LOD with and without CVD (Supplemental Tables S1 and S2). Our findings indicated that those in the EOD with CVD exhibited older age, higher SBP, and elevated LDLC levels, in addition to a greater family history of hypertension, hyperlipidemia, and CVD when compared with EOD without CVD. Similar results were observed for LOD with and without CVD.

Table 2 illustrates that the EOD group exhibited moderate obesity levels that were similar to those observed in the LOD group. The uric acid levels were similar between the two groups. However, in terms of cardiovascular risk factors such as SBP, DBP, high‐desnity lipoprotein cholesterol (HDLC), LDLC, and metabolic syndrome, the EOD group demonstrated relatively lower levels compared to the LOD group.

A total of 4949 diabetes cases were included in this study, consisting of 2908 newly diagnosed cases and 2041 previously diagnosed cases. For the purpose of therapy analysis, we focused on the 2041 diagnosed cases, of which 387 participants were classified as having EOD diabetes and 1654 participants were classified as having LOD diabetes. The average diabetes duration (from the time of diabetes diagnosis to the time of recruitment) for the EOD diabetes was 7.9 years, whereas that for the LOD diabetes was of 5.4 years. Treatment rates for EOD and LOD diabetes were 80.4% and 81.9%, respectively, indicating a lower treatment rate in China compared to other countries. It is important to note that this study was conducted in 2007–2008, before the use of glucose‐lowering drugs of cardiovascular protective agents such as sodium‐glucose cotransporter‐2 inhibitors and glucagon‐like peptide 1 agonists in China. Therefore, the glucose‐lowering drugs used in this study did not include cardiovascular protective agents, as indicated in Supplemental Table S3.

Supplemental Table S3 showed that there were no differences in oral hypoglycemic agent use between the EOD and LOD diabetes groups. However, insulin use was higher in the EOD diabetes group. Additionally, the LOD diabetes group had higher usage of antihypertensive and lipid‐lowering drugs.

**FIGURE 2:**
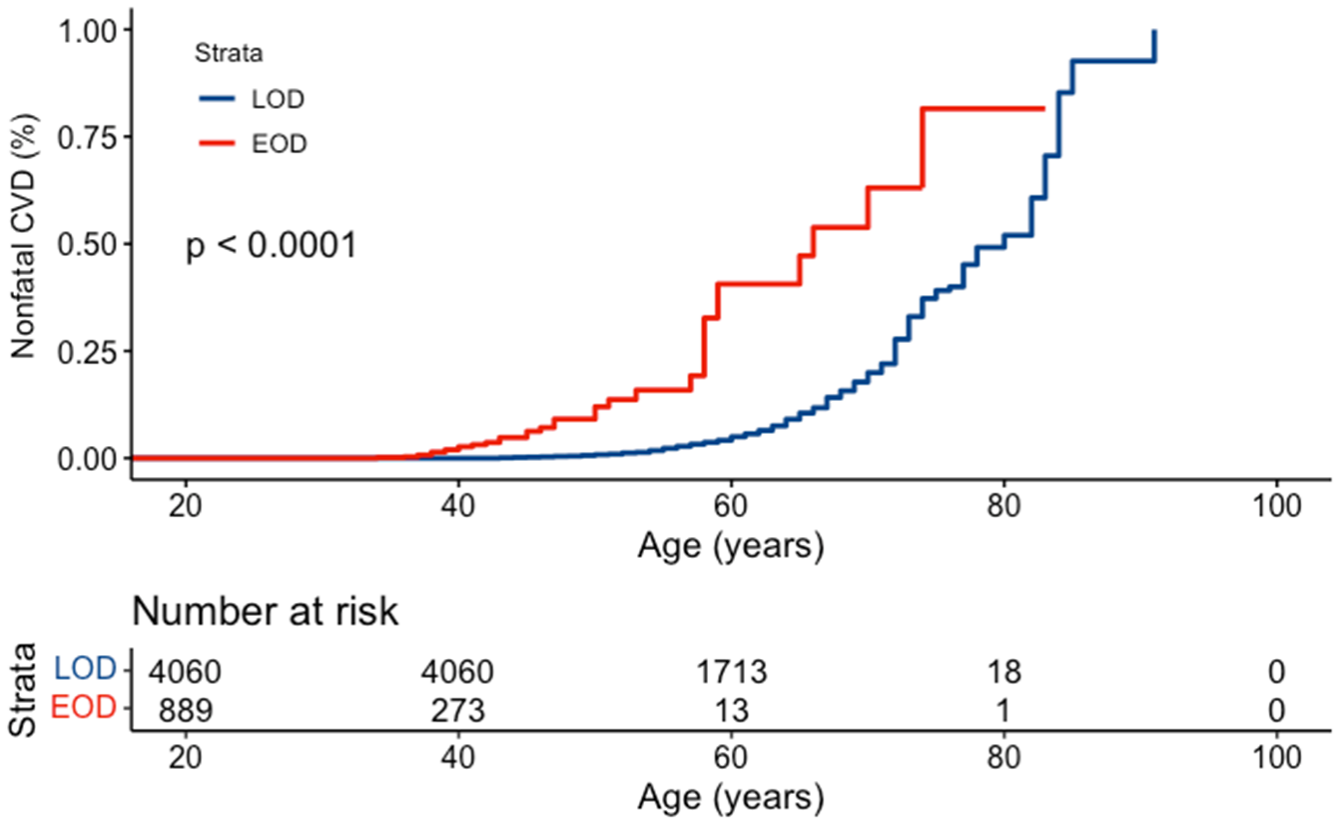
Kaplan–Meier plot of nonfatal CVD in the EOD and LOD groups. CVD, cardiovascular disease; EOD, early‐onset type 2 diabetes; LOD, late‐onset type 2 diabetes.

There were 355 nonfatal CVD events in the LOD group and 35 CVD events in the EOD group. Although the crude prevalence of nonfatal CVD was higher in the LOD group than in the EOD group (8.7% vs 3.9%), the age‐standardized prevalence of nonfatal CVD was significantly higher in the EOD group (11.4% vs 4.4%). Moreover, Kaplan–Meier plots showed that the prevalence of nonfatal CVD significantly increased in patients with EOD compared to those with LOD, starting from the age of 40 years (Figure 2).

After adjusting for age and sex, the EOD group was found to have a 2.3‐fold (95% confidence interval [CI] 1.5–3.5) increased risk of nonfatal CVD compared to the LOD group, as shown in Table 3. Furthermore, when accounting for additional factors such as smoking status, alcohol consumption, college education, regular leisure‐time physical activity, BMI, SBP, LDLC, hypoglycemic drug usage, antihypertensive drug usage, and lipid‐lowering drug usage, the EOD group was still linked to a 2.3‐fold (95% CI 1.4–3.5) increased risk of nonfatal CVD compared to that of the LOD group. However, after further adjustment for the duration of diabetes which was significant associated with nonfatal CVD, the increased risk decreased, resulting in an odds ratio of 1.8 (95% CI 1.1–2.9). The odds ratios of all risk factors related to nonfatal CVD in all models are shown in Supplemental Table S4. Moreover, we used the same models to examine the odds ratio of the EOD and LOD groups for CHD and stroke (Supplemental Tables S5 and S6). When accounting for risk factors without duration of diabetes, the EOD group had an increased risk of CHD or stroke compared with that of the LOD group. However, after accounting for the decrease in outcomes and further adjustment for duration of diabetes, the risk was not significant.

**TABLE 3:** The odds ratio of the EOD vs LOD groups for nonfatal CVD.

Models	Odds ratio (95% CI)	*p* value
Multivariable model 1		
Early vs late onset	2.3 (1.5–3.5)	<.001
Multivariable model 2		
Early vs late onset	2.3 (1.4–3.5)	.001
Multivariable model 3		
Early vs late onset	1.8 (1.1–2.9)	.023
Diabetes durations	1.0 (1.0–1.0)	.024

*Note*: Multivariate model 1 was adjusted for age and sex. Multivariate model 2 incorporated additional adjustments for smoking status, alcohol consumption, college education, regular leisure‐time physical activity, body mass index, systolic blood pressure, low‐density lipid cholesterol, family history of hyperlipidemia, hypoglycemic drug usage, antihypertensive drug usage, and lipid‐lowering drug usage. Multivariate model 3 was further adjusted for the duration of diabetes.

Abbreviations: EOD, early‐onset type 2 diabetes; LOD, late‐onset type 2 diabetes.

We conducted a sensitivity analysis among individuals with diabetes and metabolic syndrome. After adjusting for diabetes duration and other risk factors in this subgroup, the EOD group continued to exhibit a significant, although reduced, risk of nonfatal CVD with an odds ratio of 2.0 (95% CI 1.2–3.5) (Table 4). The odds ratios of all risk factors related to nonfatal CVD in all models are shown in Supplemental Table S7.

**TABLE 4:** The odds ratio of EOD vs LOD for nonfatal CVD in diabetes with metabolic syndrome.

Models	Odd ratio (95%CI)	*p* value
Multivariable model 1		
Early vs late onset	2.8 (1.7–4.4)	<0.001
Multivariable model 2		
Early vs late onset	2.6 (1.6–4.2)	<0.001
Multivariable model 3		
Early vs late onset	2.0 (1.2–3.5)	0.010
Diabetes durations	1.0 (1.0–1.1)	0.041

*Note*: Multivariate model 1 was adjusted for age and sex. Multivariate model 2 incorporated additional adjustments for smoking status, alcohol consumption, college education, regular leisure‐time physical activity, body mass index, systolic blood pressure, low‐density lipid cholesterol, family history of hyperlipidemia, hypoglycemic drug usage, antihypertensive drug usage, and lipid‐lowering drug usage. Multivariate model 3 was further adjusted for the duration of diabetes.

Abbreviations: EOD, early‐onset type 2 diabetes; LOD, late‐onset type 2 diabetes.

**TABLE 1:** Comparative baseline characteristics and glucose levels among EOD and LOD groups.

Characteristics	EOD (diagnosed age < 40 years)	LOD (diagnosed age ≥ 40 years)	*P* value
Participants	889	4060	
Females (%)	420 (47.2%)	2308 (56.8%)	<.001
Age (years)	37.3 (8.3)	57.8 (9.2)	<.001
Rural (%)	274 (30.8%)	1199 (29.5%)	.471
Cigarette smoking (%)	282 (31.7%)	1144 (28.2%)	.038
Consumption of alcohol (%)	256 (29.0%)	783 (19.4%)	<.001
Regular leisure‐time physical activity (%)	285 (32.4%)	1884 (46.7%)	<.001
College education (%)	248 (27.9%)	498 (12.3%)	<.001
Family income			.011
≤ 10 000 CNY	275 (34.0%)	1497 (39.2%)	
10 000–30 000 CNY	362 (44.7%)	1623 (42.5%)	
>30 000 CNY	173 (21.4%)	695 (18.2%)	
Family history of diabetes (%)	286 (32.2%)	921 (22.7%)	<.001
Family history of hypertension (%)	358 (40.3%)	1604 (39.5%)	.702
Family history of hyperlipidemia (%)	146 (16.4%)	394 (9.7%)	<.001
Family history of CVD (%)	163 (18.3%)	805 (19.8%)	.332
Mean FPG (mmol/L)	8.4 (3.3)	7.9 (2.7)	<.001
FPG (%)			<.001
<6.1 mmol/L	195 (21.9%)	965 (23.8%)	
6.1–7.0 mmol/L	98 (11.0%)	726 (17.9%)	
7.0–9.0 mmol/L	341 (38.4%)	1381 (34.0%)	
≥9.0 mmol/L	255 (28.7%)	988 (24.3%)	
Mean PG2h (mmol/L)	13.6 (5.9)	14.1 (5.1)	.011
PG2h (%)			<.001
<7.8 mmol/L	160 (18.0%)	379 (9.4%)	
7.8–11.1 mmol/L	120 (13.5%)	528 (13.1%)	
≥11.1 mmol/L	607 (68.4%)	3131 (77.5%)	

*Note*: Data are presented as means (SD) or *n* (%). *P* values were derived from the 𝜒^2^ test or Student's *t* test.

Abbreviations: CNY=China Yuan; CVD, cardiovascular disease; EOD, early‐onset type 2 diabetes; FPG = fasting plasma glucose; LOD, late‐onset type 2 diabetes; PG2h = venous plasma glucose concentration 2 h after standard meal test or 75 g oral glucose load.

**TABLE 2:** The cardiovascular risk factors among the EOD and LOD groups.

Characteristics	EOD	LOD	*p* value
Participants	889	4060	
Mean BMI (Kg/$m^{2}$)	25.6 (4.1)	25.7 (3.6)	.755
BMI			.044
<18.5 Kg/$m^{2}$	23 (2.6%)	66 (1.6%)	
18.5–24 Kg/$m^{2}$	289 (32.7%)	1280 (31.6%)	
24–28 Kg/$m^{2}$	341 (38.6%)	1728 (42.7%)	
≥28 Kg/$m^{2}$	230 (26.0%)	975 (24.1%)	
Waist‐to‐hip ratio	0.88 (0.1)	0.89 (0.1)	.001
Central obesity (%)	447 (50.3%)	2119 (52.2%)	.319
SBP (mm Hg)	123.8 (18.5)	135.2 (20.7)	<.001
DBP (mm Hg)	80.8 (12.1)	82.6 (11.5)	<.001
Hypertension (%)	154 (17.3%)	1545 (38.1%)	<.001
Total cholesterol (mmol/L)	4.9 (1.1)	5.1 (1.0)	<.001
Triglycerides (mmol/L)	2.1 (1.5)	2.1 (1.4)	.735
HDLC (mmol/L)	1.27 (0.3)	1.29 (0.3)	.035
LDLC (mmol/L)	2.88 (0.9)	3.04 (0.9)	<.001
UA (μ𝑚𝑜𝑙/L)	227.9 (151.1)	239.0 (144.3)	.179
Metabolic syndrome (%)	546 (61.4%)	3037 (74.8%)	<.001

*Note*: Data are presented as means (SD) or *n* (%). *P* values were derived from the 𝜒^2^ test or Student's *t* test.

Abbreviations: BMI, body mass index; DBP, diastolic blood pressure; EOD, early‐onset type 2 diabetes; HDLC, high‐density lipid cholesterol; LDLC, low‐density lipid cholesterol; LOD, late‐onset type 2 diabetes; SBP, systolic blood pressure; UA, uric acid.

## 7 DISCUSSION

In this large, multicenter, cross‐sectional, population‐based study conducted in China, 18.0% of patients with diabetes were classified as EOD. After adjusting for diabetes duration and other risk factors, individuals with EOD had a higher risk of nonfatal CVD than those with LOD. Among patients with diabetes and metabolic syndrome, EOD presented a higher risk of nonfatal CVD, with an odds ratio of 1.9 (95% CI 1.1–3.3).

Numerous studies have documented a consistent increase in the prevalence of EOD worldwide, with notable examples found in the United Kingdom,^19^, ^20^ the United States,^21^ and Hong Kong.^22^ In mainland China, the rate of EOD in the general population has increased from 1.0% in 1997 to 5.7% in 2013.^3^, ^9^ Our study revealed that 18.0% of patients with diabetes experienced EOD, indicating that the condition has reached pandemic levels in the younger population. Prompt and effective preventative measures targeting young individuals are crucial for mitigating the impact of this growing health crisis.

In our study, EOD was more commonly observed in males and was associated with a higher prevalence of smoking, alcohol consumption, irregular leisure‐time physical activity, and family history of diabetes and hyperlipidemia. Studies on Asian populations have consistently reported that EOD is more likely to affect males with a family history of diabetes.^14^, ^23^ However, some studies have reported contrasting results, suggesting that EOD is more prevalent among females in European and American populations.^19^, ^24^ The possible reasons may be due to the difference in the definition of EOD in studies or due to difference in living environment. Our study revealed that compared to LOD, EOD was associated with lower blood pressure, total cholesterol, HDLC, LDLC, a smaller proportion of chronic kidney disease, and metabolic syndrome, while maintaining a similar BMI, which is consistent with findings from new EOD research.^25^ These risk factors for nonfatal CVD in EOD were relatively low, primarily due to the younger age of these patients. Overall, we presented a more comprehensive demonstration of the baseline characteristics of EOD and LOD.

In our analysis of patients with type 2 diabetes, we observed a higher prevalence of insulin use in individuals with EOD diabetes (22.5%) compared to those with LOD diabetes (14.3%). These findings suggest that beta cell deterioration occurs at a faster rate in EOD, possibly indicating a distinct pathogenesis between the two forms of the disease. Our results are consistent with those reported in the progressive beta cell in type 2 diabetes.^26^

It has been suggested that EOD carried a greater risk of cardiovascular complications compared to LOD, with complications developing and progressing more quickly.^27^, ^28^, ^29^, ^30^ In Taiwan, a prospective study revealed that individuals diagnosed with EOD before the age of 45 faced a 3.8‐fold increased risk of developing macrovascular complication compared to those diagnosed after the age of 45 years.^31^ Similarly, data from a predominantly white population demonstrated that patients diagnosed before the age of 45 had a 14‐fold higher risk of developing myocardial infarction than the control group.^32^ These findings are consistent with our results, which indicate a significantly increased risk of nonfatal CVD in patients with EOD.

There have been multiple epidemiological studies conducted to explore whether the heightened risk of complications linked with EOD is a result of prolonged exposure to the condition.^33^, ^34^ In a prospective study in Hong Kong, the elevated risk of cardiovascular and renal complications associated with EOD has been linked to a longer disease duration.^13^ Similarly, a cross‐sectional study in China hospital found an increased risk of nonfatal CVD primarily due to the duration of diabetes.^14^ In our study, we find that duration of diabetes is an important risk factors to increased risk of non‐fatal CVD. What is more, the results also suggest that other risk factors, such as the genetic risk of CVD,^35^ may contribute to nonfatal CVD in the EOD group and should be studied further.

Our study has multiple strengths, including its nationwide scope that helps mitigate potential bias. Furthermore, we gathered a diverse array of clinical variables to provide a comprehensive representation of EOD characteristics. Nonetheless, we also acknowledge several limitations. First, the cross‐sectional study design may have overlooked competing risks of death. Second, we did not systematically screen for underlying autoimmune diabetes, including latent autoimmune diabetes in adults (LADA) and patients with type 1 diabetes. However, the prevalence of LADA and type 1 diabetes remains relatively low. Third, our study relied on self‐reported nonfatal CVD events, which may be subject to recall bias. Fourth, our cross‐sectional study did not consider the duration of exposure to all cardiovascular risk factors. Additionally, we do not have data on the glycemic status of newly diagnosed diabetes in previous years, including whether their levels were within the normal or prediabetic range, or the possibility of undiagnosed diabetes.

## 8 CONCLUSIONS

Our comprehensive analysis of EOD and LOD characteristics revealed that EOD increases the risk of nonfatal CVD compared to LOD, even after adjusting for diabetes duration and other risk factors. Furthermore, these findings were consistent in patients with diabetes and metabolic syndrome, highlighting the importance of prevention and management strategies for patients with EOD.

## AUTHOR CONTRIBUTIONS

Fei Chen and Bo Zhang participated in the design, and analysis, and wrote the manuscript. Zhaojun Yang, Yao Wang, and Wenying Yang contributed to the data collection. Liping Yu, Shuo Xie, Zhaoqing Li, Ruifen Deng, Xian Jin, and Yifan He. took part in the data analysis. Wenying Yang and Bo Zhang designed and supervised the study. All authors edited, reviewed, and approved the final version of the manuscript. Bo Zhang is the guarantor of this work and, as such, had full access to all the data in the study and takes responsibility for the integrity of the data and the accuracy of the data analysis.

## FUNDING INFORMATION

This study was supported by National High Level Hospital Clinical Research Funding (2022‐NHLHCRF‐YS‐01).

## CONFLICT OF INTEREST STATEMENT

All authors declare that they have no competing interests.

## CONSENT TO PARTICIPATE

Written informed consent was obtained from all study participants.

## CONSENT FOR PUBLICATION

All authors approved the manuscript for publication.

## Supporting information


**Data S1.** Supporting Information.

## Data Availability

The data set analyzed in the current study was available from the corresponding author upon reasonable request.
