# Supplementary material for: Cardiovascular disease risk in early‐onset vs late‐onset type 2 diabetes in China: A population‐based cross‐sectional study
Source: J Diabetes. 2023 Nov 6;16(2):e13493. doi: 10.1111/1753-0407.13493 (PMC10859311; doi:10.1111/1753-0407.13493)
Supplement: Supplementary file 1 — Data S1. Supporting Information. [file JDB-16-e13493-s001.docx]

Supplemental Material

Supplemental Table S1 Comparative clinical characteristic between EOD without CVD and EOD with CVD groups

| Characteristics | EOD_non_CVD | EOD_CVD | P |
| --- | --- | --- | --- |
| Participants | 854 | 35 |  |
| Females (%) | 404 (47.31%) | 16 (45.71%) | 0.99 |
| Age (year) | 36.80 (7.90) | 48.60 (10.65) | <0.001 |
| Rural (%) | 267 (31.26%) | 7 (20.00%) | 0.22 |
| Cigarette smoking (%) | 266 (31.15%) | 16 (45.71%) | 0.103 |
| Consumption of alcohol (%) | 246 (29.01%) | 10 (28.57%) | 1 |
| Regular leisure-time physical activity (%) | 267 (31.64%) | 18 (51.43%) | 0.023 |
| College education (%) | 241 (28.22%) | 7 (20.00%) | 0.384 |
| Family income |  |  | 0.75 |
| ≤ 10,000 CNY | 266 (34.19%) | 9 (28.12%) |  |
| 10,000 - 30,000 CNY | 347 (44.60%) | 15 (46.88%) |  |
| > 30,000 CNY | 165 (21.21%) | 8 (25.00%) |  |
| Family history of diabetes (%) | 271 (31.73%) | 15 (42.86%) | 0.232 |
| Family history of hypertension (%) | 336 (39.34%) | 22 (62.86%) | 0.009 |
| Family history of hyperlipidemia (%) | 133 (15.57%) | 13 (37.14%) | 0.002 |
| Family history of CVD (%) | 0.17 (0.38%) | 0.51 (0.51%) | <0.001 |
| Mean FPG (mmol/L) | 8.38 (3.27) | 9.33 (3.19) | 0.095 |
| FPG (%) |  |  | 0.159 |
| <6.1 mmol/L | 189 (22.13%) | 6 (17.14%) |  |
| 6.1-7.0 mmol/L | 95 (11.12%) | 3 (8.57%) |  |
| 7.0-9.0 mmol/L | 331 (38.76%) | 10 (28.57%) |  |
| ≥9.0 mmol/L | 239 (27.99%) | 16 (45.71%) |  |
| Mean pg2h (mmol/L) | 13.54 (5.94) | 16.24 (3.78) | 0.008 |
| PG2h (%) |  |  | 0.007 |
| <7.8 mmol/L | 160 (18.78%) | 0 (0.00%) |  |
| 7.8-11.1mmol/L | 117 (13.73%) | 3 (8.57%) |  |
| ≥11.1 mmol/L | 575 (67.49%) | 32 (91.43%) |  |
| Mean BMI (Kg/$m^{2}$) | 25.60 (4.13) | 26.69 (3.54) | 0.125 |
| Body mass index |  |  | 0.402 |
| <18.5 Kg/$m^{2}$ | 22 (2.59%) | 1 (2.86%) |  |
| 18.5-24 Kg/$m^{2}$ | 282 (33.25%) | 7 (20.00%) |  |
| 24-28 Kg/$m^{2}$ | 326 (38.44%) | 15 (42.86%) |  |
| ≥28 Kg/$m^{2}$ | 218 (25.71%) | 12 (34.29%) |  |
| Waist-to-hip ratio (%) | 0.88 (0.08) | 0.93 (0.11) | 0.004 |
| Central obesity (%) | 429 (50.23%) | 18 (51.43%) | 1 |
| SBP (mm Hg) | 123.16 (18.36) | 138.66 (15.72) | <0.001 |
| DBP (mm Hg) | 80.60 (11.90) | 86.14 (15.89) | 0.008 |
| Hypertension (%) | 389 (45.55%) | 31 (88.57%) | <0.001 |
| Total cholesterol (mmol/L) | 4.93 (1.09) | 5.25 (1.23) | 0.091 |
| Triglycerides (mmol/L) | 2.05 (1.52) | 2.43 (1.90) | 0.153 |
| HDLC (mmol/) | 1.27 (0.32) | 1.22 (0.31) | 0.358 |
| LDLC (mmol/L) | 2.89 (0.92) | 3.04 (0.98) | 0.348 |
| UA (μ𝑚𝑜𝑙/L) | 228.42 (151.06) | 218.48 (155.27) | 0.78 |
| metabolic syndrome (%) | 515 (60.30%) | 31 (88.57%) | 0.001 |

Data are presented as means (standard deviation) or n (%). *P* values were derived from the 𝜒2 test or Student’s t-Test. CNY=China Yuan; FPG=fasting plasma glucose; PG2h=venous plasma glucose concentration 2 h after standard meal test or 75 g oral glucose load; CVD=cardiovascular disease; SBP=systolic blood pressure; DBP=diastolic blood pressure; HDLC=high-density lipid cholesterol; LDLC=low-density lipid cholesterol; UA=uric acid; EOD_non_CVD=early-onset type 2 diabetes without CVD; EOD_CVD=early-onset type 2 diabetes with CVD.

Supplemental Table S2 Comparative clinical characteristic between LOD without CVD and LOD with CVD groups

| Characteristics | LOD_non_CVD | LOD_CVD | P |
| --- | --- | --- | --- |
| Participants | 3705 | 355 |  |
| Females (%) | 2103 (56.76%) | 205 (57.75%) | 0.763 |
| Age (year) | 57.21 (9.06) | 63.51 (8.61) | <0.001 |
| Rural (%) | 1137 (30.69%) | 62 (17.46%) | <0.001 |
| Cigarette smoking (%) | 1032 (27.85%) | 112 (31.55%) | 0.157 |
| Consumption of alcohol (%) | 734 (19.89%) | 49 (13.92%) | 0.008 |
| Regular leisure-time physical activity (%) | 1682 (45.69%) | 202 (57.06%) | <0.001 |
| College education (%) | 462 (12.47%) | 36 (10.14%) | 0.233 |
| Family income |  |  | 0.141 |
| ≤ 10,000 CNY | 1381 (39.72%) | 116 (34.32%) |  |
| 10,000 - 30,000 CNY | 1470 (42.28%) | 153 (45.27%) |  |
| > 30,000 CNY | 626 (18.00%) | 69 (20.41%) |  |
| Family history of diabetes (%) | 843 (22.75%) | 78 (21.97%) | 0.788 |
| Family history of hypertension (%) | 1,439 (38.84%) | 165 (46.48%) | 0.006 |
| Family history of hyperlipidemia (%) | 347 (9.37%) | 47 (13.24%) | 0.024 |
| Family history of CVD (%) | 0.19 (0.39%) | 0.26 (0.44%) | 0.002 |
| Mean FPG (mmol/L) | 7.97 (2.74) | 7.72 (2.56) | 0.098 |
| FPG (%) |  |  | 0.4 |
| <6.1 mmol/L | 880 (23.75%) | 85 (23.94%) |  |
| 6.1-7.0 mmol/L | 654 (17.65%) | 72 (20.28%) |  |
| 7.0-9.0 mmol/L | 1,258 (33.95%) | 123 (34.65%) |  |
| ≥9.0 mmol/L | 913 (24.64%) | 75 (21.13%) |  |
| Mean pg2h (mmol/L) | 14.14 (5.14) | 14.15 (5.07) | 0.99 |
| PG2h (%) |  |  | 0.441 |
| <7.8 mmol/L | 341 (9.26%) | 38 (10.73%) |  |
| 7.8-11.1mmol/L | 477 (12.95%) | 51 (14.41%) |  |
| ≥11.1 mmol/L | 2,866 (77.80%) | 265 (74.86%) |  |
| Mean BMI (Kg/$m^{2}$) | 25.65 (3.64) | 26.08 (3.70) | 0.031 |
| Body mass index |  |  | 0.258 |
| <18.5 Kg/$m^{2}$ | 64 (1.73%) | 2 (0.56%) |  |
| 18.5-24 Kg/$m^{2}$ | 1,174 (31.77%) | 106 (29.94%) |  |
| 24-28 Kg/$m^{2}$ | 1,576 (42.65%) | 152 (42.94%) |  |
| ≥28 Kg/$m^{2}$ | 881 (23.84%) | 94 (26.55%) |  |
| Waist-to-hip ratio (%) | 0.89 (0.07) | 0.90 (0.07) | 0.764 |
| Central obesity (%) | 1,940 (52.36%) | 179 (50.42%) | 0.52 |
| SBP (mm Hg) | 134.69 (20.85) | 140.65 (18.69) | <0.001 |
| DBP (mm Hg) | 82.62 (11.51) | 81.91 (11.37) | 0.266 |
| Hypertension (%) | 2,575 (69.50%) | 310 (87.32%) | <0.001 |
| Total cholesterol (mmol/L) | 5.12 (1.03) | 5.19 (1.17) | 0.219 |
| Triglycerides (mmol/L) | 2.05 (1.43) | 2.06 (1.26) | 0.923 |
| HDLC (mmol/) | 1.29 (0.34) | 1.27 (0.32) | 0.268 |
| LDLC (mmol/L) | 3.06 (0.89) | 3.18 (1.04) | 0.01 |
| UA (μ𝑚𝑜𝑙/L) | 238.38 (143.00) | 246.30 (158.15) | 0.5 |
| metabolic syndrome (%) | 2,740 (73.95%) | 297 (83.66%) | <0.001 |

Data are presented as means (standard deviation) or n (%). *P* values were derived from the 𝜒2 test or Student’s t-Test. CNY=China Yuan; FPG=fasting plasma glucose; PG2h=venous plasma glucose concentration 2 h after standard meal test or 75 g oral glucose load; CVD=cardiovascular disease; SBP=systolic blood pressure; DBP=diastolic blood pressure; HDLC=high-density lipid cholesterol; LDLC=low-density lipid cholesterol; UA=uric acid; LOD_non_CVD=late-onset type 2 diabetes without CVD; LOD_CVD=late-onset type 2 diabetes with CVD.

Supplemental Table S3 The therapies used in the early-onset diagnosed diabetes and the late-onset diagnosed diabetes groups

| Characteristics | Early-onset diagnosed diabetes | Late-onset diagnosed diabetes | *P* |
| --- | --- | --- | --- |
| Participants | 387 | 1,654 |  |
| Diabetes duration (year) | 7.9(9.2) | 5.4(5.0) | <0.001 |
| Received hypoglycemic drugs (%) | 311(80.4%) | 1,355(81.9%) | 0.522 |
| Biguanides (%) | 176(45.5%) | 777(47.0%) | 0.634 |
| Sulfonylureas (%) | 106(27.4%) | 453(27.4%) | 1 |
| Alpha-glucosidase inhibitors (%) | 21(5.4%) | 90(5.4%) | 1 |
| Thiazolidinediones (%) | 10(2.6%) | 32(1.9%) | 0.541 |
| Insulin (%) | 87(22.5%) | 237(14.3%) | <0.001 |
| Antihypertensive drugs (%) | 74(19.1%) | 623(37.7%) | <0.001 |
| Lipid-lowering drugs (%) | 28(7.2%) | 185(11.2%) | 0.028 |

Supplemental Table S4 The odds ratio of risk factors on non-fatal CVD in diabetes individuals

| Variables | Multivariate Model 1 | Multivariate Model 2 | Multivariate Model3 |
| --- | --- | --- | --- |
| Age | 1.09(1.07–1.1) | 1.07(1.06–1.09) | 1.07(1.05–1.08) |
| Sex | 0.97(0.78–1.2) | 1.06(0.79–1.42) | 1.04(0.78–1.4) |
| Smoking status | NA | 1.63(1.21–2.21) | 1.62(1.2–2.2) |
| Alcohol consumption | NA | 0.82(0.58–1.15) | 0.82(0.58–1.16) |
| College education | NA | 0.87(0.59–1.24) | 0.86(0.59–1.23) |
| Regular leisure-time physical activity | NA | 1.18(0.94–1.48) | 1.17(0.94–1.47) |
| BMI | NA | 1.02(0.99–1.05) | 1.02(0.99–1.06) |
| Systolic blood pressure | NA | 1(1–1.01) | 1(1–1.01) |
| Low-density lipid cholesterol | NA | 1.05(0.93–1.18) | 1.05(0.93–1.18) |
| Family history of hyperlipidemia | NA | 1.69(1.21–2.33) | 1.67(1.2–2.31) |
| Hypoglycemic drug usage | NA | 1.48(1.17–1.86) | 1.26(0.96–1.65) |
| Antihypertensive drug usage | NA | 2.67(2.09–3.42) | 2.69(2.1–3.44) |
| Lipid-lowering drug usage | NA | 2.43(1.77-3.31) | 2.46(1.79–3.35) |
| Diabetes duration | NA | NA | 1.03(1–1.05) |
| Early versus late onset | 2.28(1.46–3.49) | 2.27(1.43–3.53) | 1.78(1.07–2.91) |

Multivariate model 1 was adjusted for age and sex. Multivariate model 2 incorporated additional adjustments for smoking status, alcohol consumption, college education, regular leisure-time physical activity, BMI, systolic blood pressure, low-density lipid cholesterol, family history of hyperlipidemia, hypoglycemic drug usage, antihypertensive drug usage, and lipid-lowering drug usage. Multivariate model 3 was further adjusted for the duration of diabetes, NA=the variable was not included in the model.

Supplemental Table S5 The odds ratio of the EOD versus LOD groups for CHD

| Models | Odd ratio (95%CI) | | *P* value |
| --- | --- | --- | --- |
| Multivariable model 1 |  |  | |
| Early versus late onset | 2.3(1.0–4.9) | 0.031 | |
| Multivariable model 2 |  |  | |
| Early versus late onset | 2.5(1.1–5.5) | 0.022 | |
| Multivariable model 3 |  |  | |
| Early versus late onset | 1.7(0.7–4.2) | 0.233 | |
| Diabetes durations | 1.0(1–1.1) | 0.071 | |

Multivariate model 1 was adjusted for age and sex. Multivariate model 2 incorporated additional adjustments for smoking status, alcohol consumption, college education, regular leisure-time physical activity, BMI, systolic blood pressure, low-density lipid cholesterol, family history of hyperlipidemia, hypoglycemic drug usage, antihypertensive drug usage, and lipid-lowering drug usage. Multivariate model 3 was further adjusted for the duration of diabetes. EOD=early-onset type 2 diabetes; LOD=late-onset type 2 diabetes.

Supplemental Table S6 The odds ratio of the EOD versus LOD groups for stroke

| Models | Odd ratio (95%CI) | | *P* value |
| --- | --- | --- | --- |
| Multivariable model 1 |  |  | |
| Early versus late onset | 2.1(1.4–3.3) | <0.001 | |
| Multivariable model 2 |  |  | |
| Early versus late onset | 2.1(1.3–3.3) | 0.002 | |
| Multivariable model 3 |  |  | |
| Early versus late onset | 1.6(1–2.7) | 0.067 | |
| Diabetes durations | 1.0(1–1.1) | 0.019 | |

Multivariate model 1 was adjusted for age and sex. Multivariate model 2 incorporated additional adjustments for smoking status, alcohol consumption, college education, regular leisure-time physical activity, BMI, systolic blood pressure, low-density lipid cholesterol, family history of hyperlipidemia, hypoglycemic drug usage, antihypertensive drug usage, and lipid-lowering drug usage. Multivariate model 3 was further adjusted for the duration of diabetes. EOD=early-onset type 2 diabetes; LOD=late-onset type 2 diabetes.

Supplemental Table S7 The odds ratio of risk factors on non-fatal CVD in diabetes with metabolic syndrome

| Variables | Multivariate Model 1 | Multivariate Model 2 | Multivariate Model3 |
| --- | --- | --- | --- |
| Age | 1.08(1.07–1.1) | 1.07(1.06–1.09) | 1.07(1.05–1.08) |
| Sex | 0.87(0.68–1.1) | 0.95(0.69–1.33) | 0.94(0.68–1.3) |
| Smoking status | NA | 1.47(1.05–2.07) | 1.46(1.04–2.05) |
| Alcohol consumption | NA | 0.8(0.54–1.18) | 0.8(0.54–1.18) |
| College education | NA | 0.79(0.51–1.17) | 0.78(0.51–1.16) |
| Regular leisure-time physical activity | NA | 1.13(0.88–1.45) | 1.13(0.88–1.45) |
| BMI | NA | 1.01(0.98–1.05) | 1.02(0.98–1.05) |
| Systolic blood pressure | NA | 1(0.99–1.01) | 1(0.99–1.01) |
| Low-density lipid cholesterol | NA | 1.05(0.92–1.2) | 1.05(0.92–1.2) |
| Family history of hyperlipidemia | NA | 1.52(1.05–2.17) | 1.51(1.04–2.15) |
| Hypoglycemic drug usage | NA | 1.6(1.24–2.05) | 1.36(1.01–1.83) |
| Antihypertensive drug usage | NA | 2.53(1.95–3.3) | 2.54(1.95–3.32) |
| Lipid-lowering drug usage | NA | 2.38(1.72–3.28) | 2.4(1.73–3.3) |
| Diabetes duration | NA | NA | 1.03(1–1.05) |
| Early versus late onset | 2.79(1.72–4.44) | 2.58(1.56–4.17) | 2.04(1.17–3.48) |

Multivariate model 1 was adjusted for age and sex. Multivariate model 2 incorporated additional adjustments for smoking status, alcohol consumption, college education, regular leisure-time physical activity, BMI, systolic blood pressure, low-density lipid cholesterol, family history of hyperlipidemia, hypoglycemic drug usage, antihypertensive drug usage, and lipid-lowering drug usage. Multivariate model 3 was further adjusted for the duration of diabetes, NA=the variable was not included in the model.
